# Using hydrophilic introducer sheath for peripheral vaECMO cannulation in highly calcified vessels: the bailout solution

**DOI:** 10.1007/s10047-020-01169-w

**Published:** 2020-04-27

**Authors:** Christopher Gaisendrees, Laura Suhr, Borko Ivanov, Jonas Wörmann, Ilija Djordjevic, Kaveh Eghbalzadeh, Wael Ahmad, Anton Sabashnikov, Thorsten Wahlers

**Affiliations:** 1 Department of Cardiothoracic Surgery, University Hospital of Cologne, Cologne, Germany; 2 Department of Vascular Surgery, University Hospital of Cologne, Cologne, Germany; 3 Department of Cardiology, University Hospital of Cologne, Cologne, Germany

## Objectives

Peripheral percutaneous vaECMO cannulation in acute situations, such as cardiopulmonary resuscitation (eCPR), can be highly demanding due to calcification and anatomical variations of the femoral vessels [1]. We introduce a method of accessing peripheral vessels utilizing a hydrophilic introducer sheath (Gore Dry Seal Flex).

## Methods

We report on a 71-year-old multimorbid male patient after OHCA, who was transferred to our center for eCPR. Despite several attempts by experienced colleagues, it was not possible to pass the femoral artery using the standard 17-Fr arterial cannula. Therefore, an 18-Fr hydrophilic introducer sheath (Gore Dry Seal Flex) was implanted, in which a 17-Fr arterial ECMO-cannula was positioned.

## Results

A 71-year-old man was transferred to the catheter laboratory under mechanical CPR, after OHCA and 5-min low-flow time. Coronary angiography showed no coronary artery stenosis. Arterial pH was 7.2 and lactate 10 mmol/L on arrival. The patient’s medical history revealed a pulmonary vein isolation procedure earlier that year, planned CRT-D implantation, and an aortic aneurysm of 5.7 cm. A severe arrhythmic event was assumed. We decided to establish a vaECMO in the heart catheter lab.

**Fig. 1:**
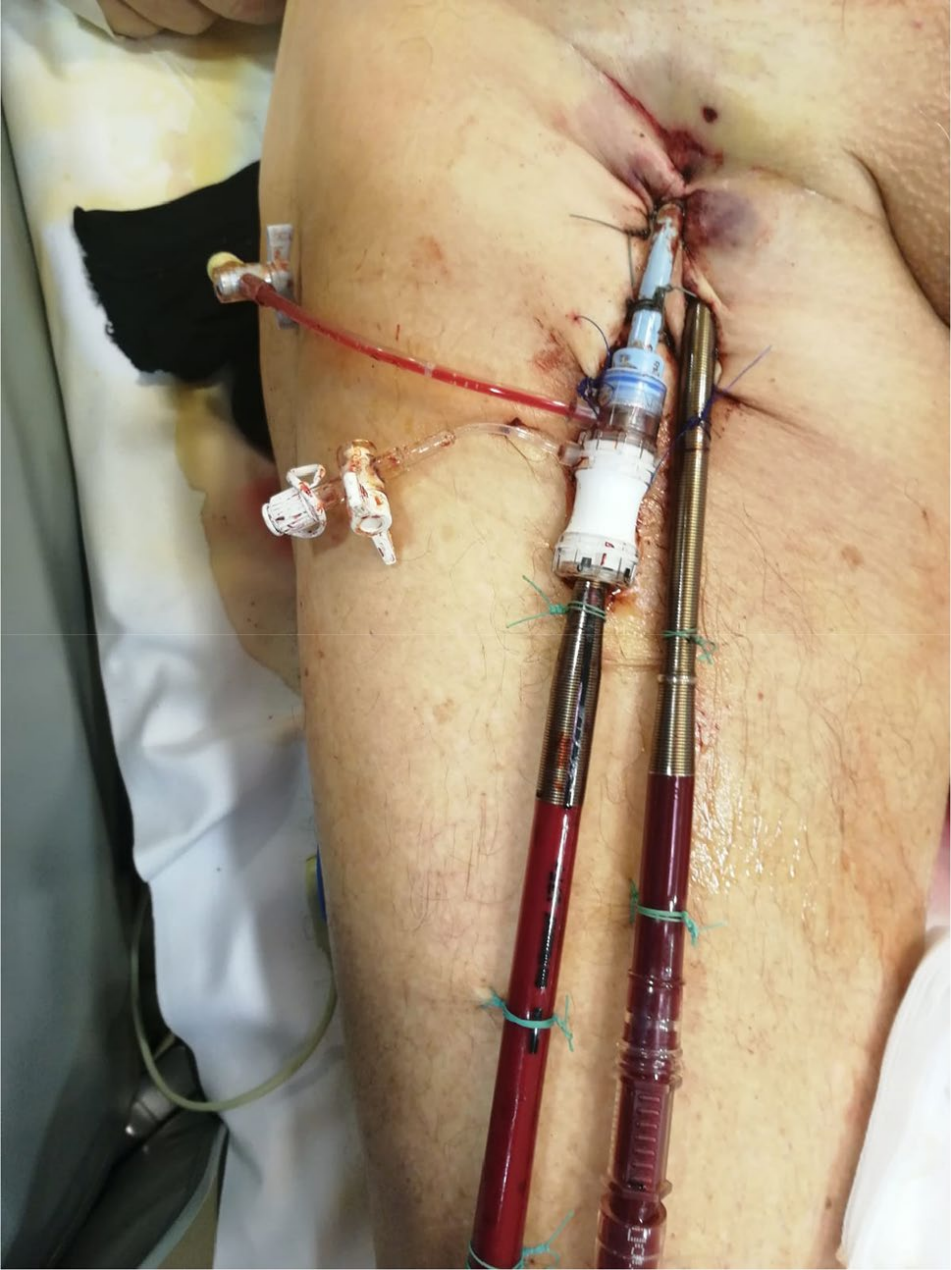
Peripheral cannulation using an 18-Fr hydrophilic introducer sheath

After placing an 8-Fr introducer sheath to the left the femoral artery, further dilatation of the vessel was impossible due to bending and snapping of the wire and dilatators. After accessing the contralateral femoral artery, the same difficulties occurred due to rigid and highly calcified vessels. As a bailout solution, an 18-Fr dry seal introducer sheath (Gore Dry Seal Flex) was easily introduced into the femoral artery and a 17-Fr arterial cannula was inserted right into the hydrophilic sheath (Fig. 1).

The Dry Seal Flex Introducer Sheath by Gore is a hydrophilic coated sheath that provides enhanced flexibility and, therefore, improved access to branched or highly calcified vessels in difficult anatomy. Sheath sizes are available from 12–26 Fr and lengths of 33–65 mm. Due to the challenging access of the peripheral arterial vessels, we decided to use a Dry Seal Introducer Sheath, to establish arterial ECMO flow.

After that, venous cannulation was successfully performed in a standard way, whereas the vein also showed extreme rigidity. A flow of 6 L/min could be achieved without any problems. During the stay in the intensive care unit, we found elevated measures of neuron-specific enolase (NSE), due to missing neurological reactions, the patient underwent cranial-CT, where edema with unfavorable prognosis was seen. Due to the severity of the cranial imaging, we decided to terminate any further therapy. In total, the ECMO-therapy was five full days with no technical problems concerning the elevated pressure of the arterial cannula through the introducer sheath.

## Conclusion

In cases of difficult access to peripheral vessels, due to anatomical variants or calcification, using a hydrophilic introducer sheath may be lifesaving and time-saving.
